# Antibiotic Consumption and Its Relationship with Bacterial Resistance Profiles in ESKAPE Pathogens in a Peruvian Hospital

**DOI:** 10.3390/antibiotics10101221

**Published:** 2021-10-08

**Authors:** Giancarlo Pérez-Lazo, Susan Abarca-Salazar, Renata Lovón, Rocío Rojas, José Ballena-López, Adriana Morales-Moreno, Wilfredo Flores-Paredes, Berenice Arenas-Ramírez, Luis Ricardo Illescas

**Affiliations:** 1 Division of Infectious Diseases, Guillermo Almenara Irigoyen National Hospital-EsSalud, Lima 15033, Peru; jose.ballena@upch.pe (J.B.-L.); adri.mmm93@hotmail.com (A.M.-M.); 2 Faculty of Infectious and Tropical Diseases, London School of Hygiene and Tropical Medicine, London WC1E 7HT, UK; susan.abarca-salazar1@student.lshtm.ac.uk; 3 Hospital Pharmacy Unit, Guillermo Almenara Irigoyen National Hospital-EsSalud, Lima 15033, Peru; renalovon@yahoo.com.pe (R.L.); rrocio10@yahoo.com (R.R.); 4 Clinical Pathology Department, Guillermo Almenara Irigoyen National Hospital-EsSalud, Lima 15033, Peru; wido.flores@gmail.com; 5 Infection Prevention and Control Unit, Guillermo Almenara Irigoyen National Hospital-EsSalud, Lima 15033, Peru; abar97@hotmail.com

**Keywords:** ESKAPE, antibiotic consumption, antimicrobial resistance, Peru

## Abstract

A descriptive design was carried out studying the correlation between antimicrobial consumption and resistance profiles of ESKAPE pathogens (*Enterococcus faecium*, *Staphylococcus aureus*, *Klebsiella pneumoniae*, *Acinetobacter baumannii*, *Pseudomonas aeruginosa*, and *Enterobacter* spp.) in a Peruvian hospital, including the surgical, clinical areas and the intensive care unit (ICU) during the time period between 2015 and 2018. There was a significant correlation between using ceftazidime and the increase of carbapenem-resistant *Pseudomonas aeruginosa* isolations (R = 0.97; *p* < 0.05) and the resistance to piperacillin/tazobactam in *Enterobacter* spp. and ciprofloxacin usage (R = 0.97; *p* < 0.05) in the medical wards. The *Pseudomonas aeruginosa* resistance to piperacillin/tazobactam and amikacin in the intensive care unit (ICU) had a significant reduction from 2015 to 2018 (67% vs. 28.6%, 65% vs. 34.9%, *p* < 0.001). These findings give valuable information about the rates and dynamics in the relationship between antibiotic usage and antimicrobial resistance patterns in a Peruvian hospital and reinforce the need for continuous support and assessment of antimicrobial stewardship strategies, including microbiological indicators and antimicrobial consumption patterns.

## 1. Introduction

*Enterococcus faecium*, *Staphylococcus aureus*, *Klebsiella pneumoniae*, *Acinetobacter baumannii*, *Pseudomonas aeruginosa* and *Enterobacter* spp. are a group of pathogens included in the ESKAPE acronym to point out the concern about this group for its ability of “escaping” the bactericidal activity of antimicrobials and therefore raise challenges to treating these infections, which no longer respond to available antibiotics not only at hospitals, but in the community [1,2,3]. The ESKAPE resistant strains are considered within the urgent or serious threat levels for antimicrobial resistance as they are associated with a high risk of mortality and high hospital costs [4,5]. The alarming rise of antimicrobial resistance has a multifactorial etiology where antibiotics’ misuse and limited infection prevention and control measures are the main known and modifiable causes [6]. Antibiotic overuse in multiple settings, including the animal, agriculture and human sectors, is one of the main and modifiable causes [7]; consequently, along with preventive and control measures, understanding the mechanisms causing resistance and antimicrobial usage dynamics in community and clinical settings will help establish more effective strategies to tackle this threat [8,9,10]. Selective pressure is a bacterial mechanism where under antibiotic presence susceptible strains disappear, facilitating the survival of intrinsically resistant species. Other mechanisms include the horizontal transfer of resistance genes, changes in cell permeability, efflux or therapeutic target and the selection of hypermutable clones [6]. The Global Point Prevalence Survey or Global-PPS is a partnership that evaluates the antimicrobial usage and bacterial resistance in 303 hospitals from 53 countries from different income categories. The 2015 report found that Latin American countries have higher rates of carbapenems, ceftriaxone and vancomycin prescriptions. At the same time, targeted antimicrobial therapy against carbapenem-resistant *Enterobacteriaceae* (CRE), extended-spectrum beta-lactamases (ESBL) and methicillin-resistant *Staphylococcus aureus* (MRSA) in inpatients was higher than in other regions [11]. The antimicrobials usage was higher on intensive care units (ICU) and transplant units than general or surgical wards. Furthermore, in this region, the multiresistant Gram-negative bacilli rate was higher than half of the total nosocomial infections reported [11,12].

Bacterial resistance represents a serious problem in Peru because over 50% of Gram-negative bacteria isolated are *Escherichia coli* and *Klebsiella pneumoniae* ESBL across different hospitalization areas, including general rooms, emergency rooms and intensive care units [13]. In response to the antimicrobial resistance challenge, some Peruvian hospitals have started the implementation of antimicrobial stewardship (AMS) programs in order to reduce the consumption of broad-spectrum antibiotics [13,14]. These response strategies include antimicrobial restriction, educational campaigns and promotion of antimicrobial profiles and algorithms, prospective audit, pre-authorized forms and monitoring empirical antimicrobial treatment flowcharts elaborated in consensus with the intensive care, infection control, pharmacy and microbiology units [13]. Other countries in the region have also added automatic and technology-enhanced monitoring strategies, still not reported in Peru [15]. This study was conducted along with the implementation of an antimicrobial stewardship program and aims to evaluate the resistance profile of the bacteria ESKAPE and to study the correlation with the consumption of antimicrobials in three hospitalization areas of a Peruvian hospital.

## 2. Results

**Figure 1:**
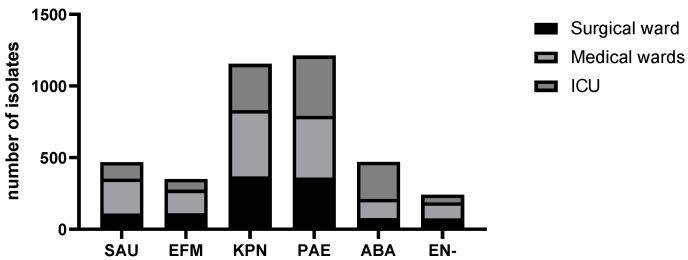
Distribution of ESKAPE pathogens by hospital areas. Abbreviations: SAU = *Staphylococcus aureus*, EFM = *Enterococcus faecium*, KPN = *Klebsiella pneumoniae*, PAE = *Pseudomonas aeruginosa*, ABA = *Acinetobacter baumannii*, EN- = *Enterobacter* spp. ICU = Intensive care unit.

Among the ESKAPE pathogens evaluated, *Klebsiella pneumoniae* (*n* = 1154) and *Pseudomonas aeruginosa* (*n* = 1212) were the most frequently isolated microorganisms. The distribution of ESKAPE pathogens by areas during the study period is shown in Figure 1.

**Figure 2:**
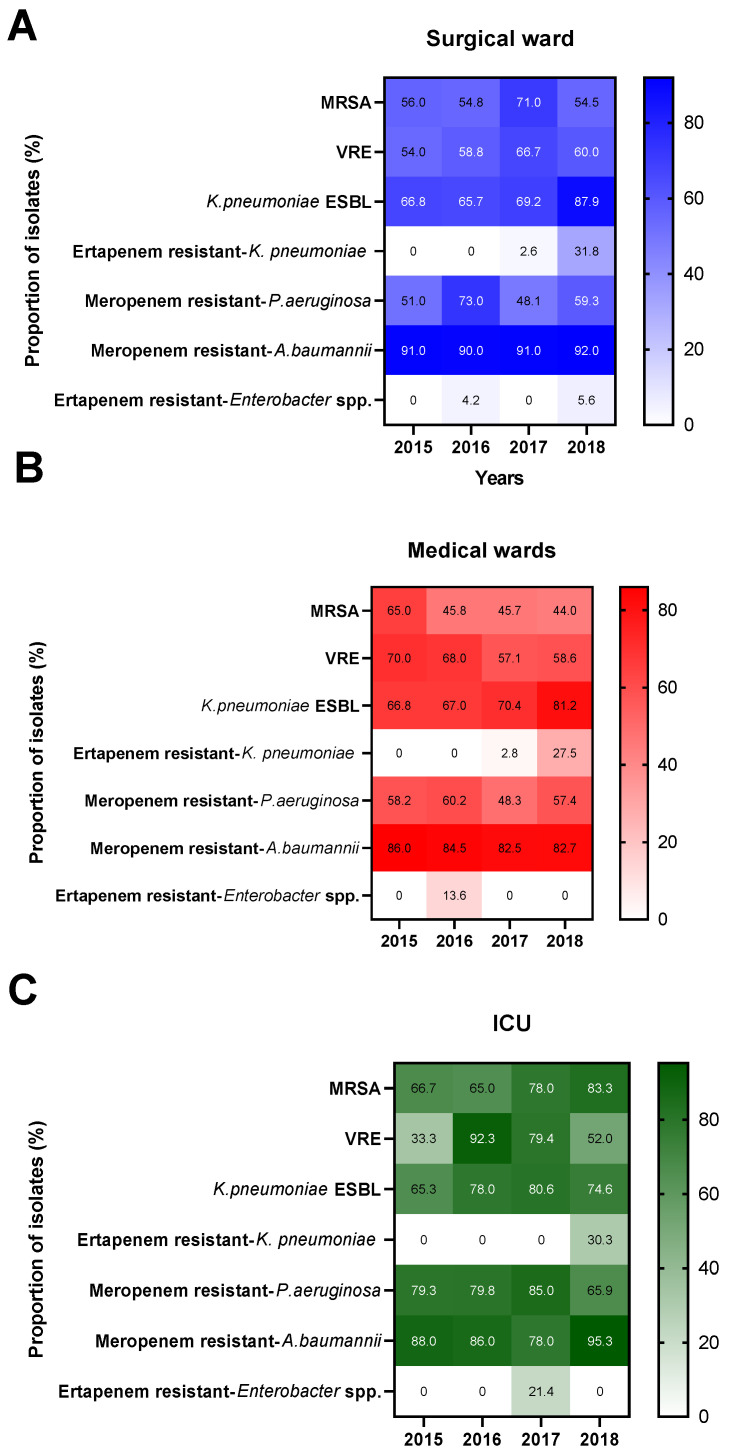
Evolution of the antimicrobial resistance rate of ESKAPE pathogens by hospital areas. (**A**). Surgical ward (**B**). Medical wards (**C**). ICU. Abbreviations: MRSA = methicillin-resistant *Staphylococcus aureus*, VRE = Vancomycin resistant *Enterococcus faecium*, ICU = Intensive care unit.

Antimicrobial resistance profiles of the evolution and the antimicrobial resistance rate of ESKAPE pathogens by wards and years are detailed in Figure 2 and Table S1. During the study period, the average rate of MRSA and vancomycin-resistant *Enterococcus faecium* (VRE) isolate exceeded 50% and 60%, respectively, in all three areas and the highest resistance rate was found in the ICU (73.3% and 64.3%). Regarding the resistance profile trend, there were no significant changes during the follow-up period. In the three study areas, the extended-spectrum beta-lactamases (ESBL) rate in *Klebsiella pneumoniae* was higher than 70%, whereas an overall increase of *Klebsiella pneumoniae* resistance to carbapenems (*p* < 0.001) and piperacillin/tazobactam (*p* < 0.05) was found in the last year of follow-up.

The carbapenem-resistant *Pseudomonas aeruginosa* rate in the surgical and medical wards was around 60%, and over 75% in the intensive care unit. A significant decrease in the resistance to piperacillin/tazobactam and amikacin was observed (*p* < 0.001) only in the ICU when the last and first year of follow-up were compared. Colistin resistance was not recorded during the study period. The resistance rate to other antimicrobials with antipseudomonal action such as ceftazidime or ciprofloxacin exceeded 50% in all the studied areas. Likewise, the rate of carbapenem-resistant *Acinetobacter baumannii* in all areas was greater than 85%. The *Acinetobacter baumannii* multiresistance profile did not vary throughout the study, and no colistin or tigecycline resistance was found in the evaluated strains. The resistance rate to third-generation cephalosporins in *Enterobacter* spp. in the surgical and medical wards exceeded 50%; however, no changes in the resistance profile trend were found in the antibiotics tested.

**Figure 3:**
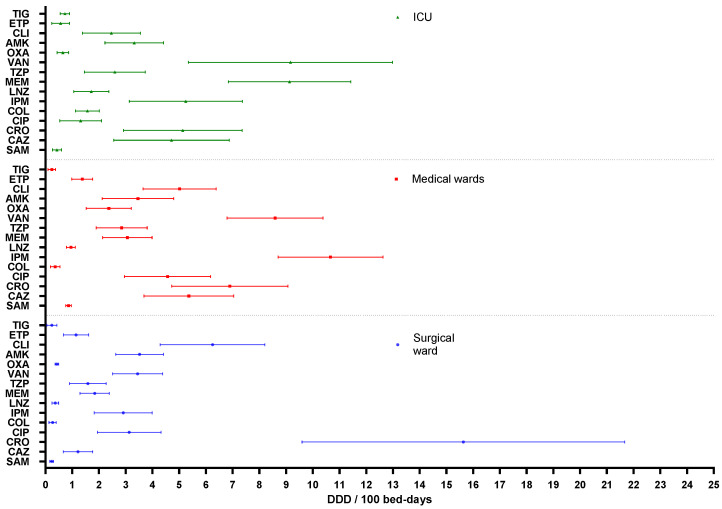
Antimicrobial consumption (mean and standard deviation during the period 2015–2018) by hospital areas. DDD = defined daily doses. AMK = amikacin, CAZ = ceftazidime, CIP = ciprofloxacin, CLI = clindamycin, COL = colistin, CRO = ceftriaxone, ETP = ertapenem, IPM = imipenem, LNZ = linezolid, MEM = meropenem, OXA = oxacillin, SAM = ampicillin/sulbactam, TIG = tigecycline, TZP = piperacillin/tazobactam, VAN = vancomycin. ICU = intensive care unit.

The relative annual consumption of each antimicrobial, the mean, the standard deviation and the percentage of change during the study period are presented in Figure 3 and Table S2. The antimicrobials that had the highest average consumption in the ICU were vancomycin (9.16 DDD/100 bed-days) and meropenem (9.13 DDD/100 bed-days). In this area, the percentage of change with respect to 2015 was higher for linezolid (+125.42%) and the usage of third-generation cephalosporins, carbapenems, vancomycin and amikacin decreased. The antimicrobial with the highest average consumption in the surgical ward was ceftriaxone (15.63 DDD/100 bed-days), followed by clindamycin (6.25 DDD/100 bed-days) and ciprofloxacin (3.13 DDD/100 bed-days). In the medical wards, the antimicrobials with the highest consumption were imipenem (10.66 DDD/100 bed-days) and vancomycin (8.59 DDD/100 bed-days). The percentage of change in antimicrobial consumption in both services was greater for ertapenem (>100%), tigecycline (>200%), colistin (>250%), meropenem (>50%) and piperacillin/tazobactam (>50%).

**Figure 4:**
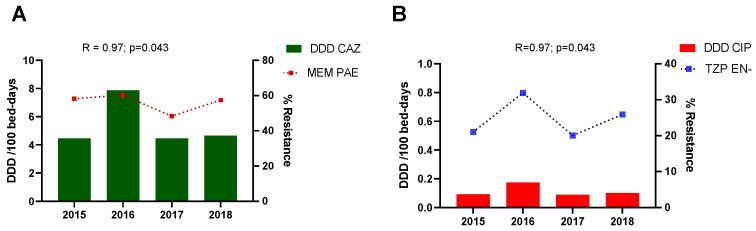
Antimicrobial consumption and correlation with resistance for specific pathogen in medical wards. (**A**). Ceftazidime consumption and resistance to meropenem *in Pseudomonas aeruginosa*. (**B**). Ciprofloxacin consumption and resistance to piperacillin*/*tazobactam in *Enterobacter* spp. DDD = defined daily doses. CAZ = ceftazidime, CIP = ciprofloxacin, MEM = meropenem, TZP = piperacillin/tazobactam.

The correlations between antimicrobial resistance and antimicrobial consumption per service and microorganism are shown in Table 1. A positive correlation between ceftazidime consumption and resistance to meropenem in *Pseudomonas aeruginosa* (R = 0.97; *p* = 0.031) (Figure 4) and between the consumption of ciprofloxacin and resistance to piperacillin/tazobactam in *Enterobacter* spp. (R = 0.97; *p* = 0.031) was found in the medical wards (Figure 4). In the surgical areas, a negative correlation was identified between the consumption of ceftazidime and resistance to carbapenems in *Acinetobacter baumannii* (R = −0.93; *p* = 0.007) and between the consumption of ciprofloxacin and resistance to carbapenems (R = −0.93; *p* = 0.007). No significant correlations were found in *Klebsiella pneumoniae*, *Staphylococcus aureus* or *Enterococcus faecium* for the selected antimicrobials in any study area.

## 3. Discussion

The analysis of the ESKAPE pathogens resistance profiles in our study showed a high level of resistance to many antibiotics. For instance, *Staphylococcus aureus* presented resistance to oxacillin above 50% and *Enterococcus faecium* at 90% to vancomycin. The rate of *Klebsiella pneumoniae* ESBL was between 65% and 87% and resistance to carbapenems was around 30% in the last year of the study. *Pseudomonas aeruginosa* presented above 50% of resistance to carbapenems with even a higher level in the ICU (85%) and *Acinetobacter baumannii* showed around 90% of resistance to meropenem. In addition to the appearance of carbapenem-resistant *Klebsiella pneumoniae* isolates during the last year of study, 13% of *Enterobacter* spp. isolates showed resistance to carbapenem.

A correlation between some ESKAPE pathogens resistance and antimicrobial consumption in the medical wards was also found, such as a positive correlation between the consumption of ceftazidime and the resistance to meropenem in *Pseudomonas aeruginosa*, and between ciprofloxacin usage and *Enterobacter* spp. resistance to piperacillin/tazobactam.

The high level of bacterial resistance in this study is consistent with the 2020 Pan American Health Organization (PAHO) report [16] that found that Peruvian isolates had the highest regional rate of carbapenem-resistant *Pseudomonas aeruginosa* and carbapenem-resistant *Acinetobacter baumannii* with 69% and 89%, respectively, in 2016. In contrast, the carbapenem-resistant *Klebsiella pneumoniae* rate in Peru was 8% compared to 16% in Colombia and 24% in Ecuador the same year. The increasing carbapenem-resistant *Klebsiella pneumoniae* (CRE) outbreaks in Peru [17,18] were associated with higher mortality rates than the infections caused by non-carbapenem-resistant strains, thus necessitating the establishment of multidisciplinary and multilevel strategies to control this threat [19].

Antimicrobial consumption is reported as one of the leading causes of antimicrobial resistance in clinical settings, where factors such as prior antibiotic use, prior hospitalization and long antibiotic treatment have shown a significant association [20,21]. However, a linear relationship with a particular class of antimicrobial drugs has not been found yet [22,23]. Despite the limitations of this study to report causal association, our exploratory analysis did not find a significant correlation with a particular antimicrobial or pathogen resistance pattern. Furthermore, the increasing number ESKAPE bacteria with different resistance patterns isolated from community samples has raised alarms about the factors associated and the influence of the antimicrobial and prescription usage in community settings [3,24].

Although the non-significant correlation between the ESKAPE pathogens resistance patterns and specific antimicrobials was consistent with other reports [25,26], significant reduction in the resistance rate of piperacillin/tazobactam and amikacin in *Pseudomonas aeruginosa* was observed in the ICU. Likewise, the rate of antipseudomonal drugs used in the ICU (ciprofloxacin, ceftazidime and imipenem) showed a decreasing trend (>50%). This may suggest that a decrease in the general consumption of an antimicrobial group contributes to modifications in the resistance profile [27]. 

A positive correlation was observed between the consumption of ceftazidime and resistance to meropenem in medical wards; similarly, Plüss-Suard et al. reported an association between broad-spectrum antimicrobials consumption and the development of multidrug-resistance in *Pseudomonas aeruginosa* [28]. Although a significant correlation between the consumption of carbapenems and carbapenem-resistant *Pseudomonas aeruginosa* was not found, a patient-centered study reported a significant relation between ertapenem usage and the appearance of strains resistant to carbapenems and ureidopenicillins [29]. In contrast, some ecological studies have suggested that ertapenem consumption (group I carbapenem without antipseudomonal activity) is not related to the increase in resistance of antipseudomonal carbapenems in *Pseudomonas aeruginosa* [30,31]. Although increasing consumption of ertapenem and tigecycline was a strategy applied to reduce carbapenem usage and pseudomonal resistance in the ICU, no changes were observed in the *Klebsiella pneumoniae* profile [32,33].

A negative correlation was identified between the consumption of ciprofloxacin and resistance to carbapenems in *Acinetobacter baumannii*. These results were in contrast with other studies that showed a positive correlation between quinolone intake with the incidence of imipenem-resistant *Acinetobacter baumannii* [34]. However, it has been suggested that the impact of antimicrobial consumption in the *Acinetobacter baumannii* resistant profile has low, if any, impact compared with other Gram-negative pathogens [35].

This study has some limitations. First, an individual approach was used and other risk factors, such as previous antibiotic usage, previous hospitalization or long antibiotic courses, were not included in the analysis. An ecological approach might contribute better to understand the dynamics of resistance patterns and microbial genetic, host and environmental interactions in different hospitalization areas. Second, only phenotypic resistance profiles were used, which probably had a combination of genetic resistance mechanisms; thereby, the antimicrobial resistance trend reported cannot be considered the cause of the specific use of any antibiotic in our hospital. Third, considering the observational design of this study and the high rate of antimicrobial resistance reported in clinical settings and even at the community level in Peru, the influence of non-nosocomial factors in the evolution of the antimicrobial-resistant pattern cannot be ruled out. Another possible confusing factor that we did not add into the analysis was the influence of the implementation of the antimicrobial stewardship program in the number of cultures requested as well as the patients’ characteristics and outcomes in the different wards included. Finally, the study cohort only involved patients from a referral hospital of the social health insurance system in Peru and a short follow-up period. Therefore, these results may not be applicable to all clinical settings in Peru.

Considering the potential positive impact of the antimicrobial stewardship strategies in reducing antimicrobial consumption and antimicrobial resistance rates, monitoring, reporting and establishing AMS strategies are essential to improving health care, especially in settings with a high level of antimicrobial resistance. The AMS program in our hospital started during the study period (midterm 2017) and the follow-up study was carried out from 2016 to 2018. Therefore, future studies should include a longer follow-up period with a robust statistic design that could help to estimate the precise impact of the antimicrobial consumption and AMS strategies in the ESKAPE antimicrobial resistance profile.

## 4. Materials and Methods

This is an observational and retrospective study that evaluates the relationship between the consumption of antimicrobials and the resistance profiles of ESKAPE pathogens in the clinical, surgical wards and the ICU of the Guillermo Almenara Irigoyen National Hospital (HNGAI), a referral hospital of the social health insurance system in Peru. This study was approved by the hospital ethics committee, and was conducted from 2015 to 2018.

### 4.1. Antimicrobial Resistance Profiles

Antimicrobial resistance profiles were obtained using the WHONET 5.6 database. We excluded epidemiological surveillance samples, internal and external quality controls and duplicate results from the same patient samples that were taken in fewer than 30 days. Non-susceptible strains from *Enterococcus faecium*, *Staphylococcus aureus*, *Klebsiella pneumoniae*, *Acinetobacter baumannii*, *Pseudomonas aeruginosa* and *Enterobacter* spp. were interpreted according to the Clinical & Laboratory Standards Institute (CLSI) cut-off points and the respective version of the year in which each sample was isolated. Antimicrobial susceptibility was determined by the automated MicroScan Walk Away 96 system.

### 4.2. Antimicrobial Consumption

The antimicrobial consumption data were obtained from the electronic database of the pharmacy department and evaluated by hospital areas. The formula for antimicrobial consumption was based on the Anatomical, Therapeutic, and Chemical (ATC) classification system and the 2019 World Health Organization (WHO) report that defines daily doses as DDD/100 bed-days [36]. The statistic unit of the hospital report was the source to calculate the number of hospital beds and monthly occupancy rate in the medical areas, including clinical speciality wards and the surgical areas (surgical units and subspecialties). Pediatrics, neonatology, nephrology units and emergency room were excluded. The DDDs reference values were ceftriaxone (2 g), ceftazidime (4 g), vancomycin (2 g), ciprofloxacin (0.5 g), ampicillin/sulbactam (6 g), clindamycin (1.8 g), oxacillin (0.5 g), amikacin (0.05 g), imipenem/cilastatin (2 g), meropenem (2 g), ertapenem (1 g), piperacillin/tazobactam (14 g), linezolid (1.2 g), tigecycline (0.1 g), colistin sulfomethate sodium [0.240g~3MU].

### 4.3. Antimicrobial Consumption-Bacterial Resistance Correlation

Correlations between the following antimicrobial drugs and bacterial resistance profiles were considered: *Staphylococcus aureus* and oxacillin, ciprofloxacin, clindamycin; *Enterococcus faecium* and vancomycin; *Klebsiella pneumoniae* and CF3G, carbapenems, ciprofloxacin, amikacin, piperacillin/tazobactam; *Pseudomonas aeruginosa* and ceftazidime, piperacillin/tazobactam, carbapenems, ciprofloxacin, amikacin; *Acinetobacter baumannii* and imipenem/cilastatin, meropenem, piperacillin/tazobactam, CF3G, ciprofloxacin; *Enterobacter* spp. and CF3G, piperacillin/tazobactam, carbapenems, amikacin.

### 4.4. Statistical Analysis

Descriptive analysis of antimicrobial consumption expressed in DDD/100 bed-days for each service was carried out, indicating the average consumption of the four years of study and its respective standard deviation. To study the consumption evolution during the study period, the percentage of change (increase or reduction) was calculated by subtracting the consumption data (DDD/100 beds-days) from 2018 compared to 2015, dividing by the consumption in the first-year of study, and multiplying the result by 100 [37]. An increasing or decreasing trend was considered when the percentage of change with respect to the previous selected year by therapeutic group varied more than 50% [38].

To evaluate the evolution of the bacterial resistance profile, the proportions (chi-square) between the first and last year of follow-up were tested. Spearman’s non-parametric test and its probability value were used to study the correlation between antimicrobial consumption and bacterial resistance. The statistical analyzes were performed with R software version 3.4.4 and a *p* ≤ 0.05 was considered statistically significant. Graphics were made with the GraphPad Prism 9.0.0.

## 5. Conclusions

The present study examined the dynamics of antimicrobial consumption and its possible impact on the antimicrobial resistance of the ESKAPE pathogens group in a referral hospital in Peru. A significant correlation between the consumption of ceftazidime and resistance to meropenem in *Pseudomonas aeruginosa* and between the consumption of ciprofloxacin and resistance to piperacillin/tazobactam in *Enterobacter* spp. was found only in the medical wards, which might suggest an influence of antimicrobial usage in the high rates of antimicrobial resistance in this hospital area. This study has room for further improvement; future work should include a longer follow-up period with a robust statistical design to gain a better understanding of the impact of the antimicrobial consumption and the AMS strategies in the ESKAPE antimicrobial resistance profile. Finally, these findings highlight the importance of improving AMS strategies moving from general to specific antimicrobial usage monitoring measures.

## Figures and Tables

**Table 1:** Results of the Spearman correlation demonstrating the relationship between the frequency of strains resistant to a particular antimicrobial and the consumption of antimicrobials that potentially exert selection pressure divided by services.

Surgical Ward			Statistical Values		Medical Wards			Statistical Values		ICU			Statistical Values	
Antibiotic Consumption	Bacterial Resistance		R	*p*	Antibiotic Consumption	Bacterial Resistance		R	*p*	Antibiotic Consumption	Bacterial Resistance		R	*p*
**TZP (↑)**	TZP	PAE	0.95	0.051	**TZP (↑)**	TZP	PAE	0.80	0.200	**TZP (↓)**	TZP	PAE	0.32	0.683
	TZP	EN-	0.95	0.051		TZP	EN-	0.16	0.834		TZP	EN-	−0.83	0.167
	IPM	KPN	0.23	0.772		IPM	KPN	0.95	0.051		IPM	KPN	0.00	1.000
	MEM	KPN	0.23	0.772		MEM	KPN	0.95	0.051		MEM	KPN	0.00	1.000
	MEM	PAE	0.80	0.200		MEM	PAE	0.79	0.201		MEM	PAE	0.00	1.000
**MEM (↑)**	MEM	KPN	−0.06	0.944	**MEM (↑)**	MEM	KPN	0.63	0.367	**IPM (↓)**	IPM	KPN	−0.77	0.225
	TZP	EN-	0.95	0.051		TZP	EN-	0.36	0.635		IPM	PAE	−0.40	0.600
	TZP	KPN	0.57	0.431		TZP	KPN	0.85	0.153		MEM	KPN	−0.77	0.225
	MEM	PAE	0.95	0.051		MEM	PAE	0.89	0.102		TZP	KPN	−0.40	0.600
	MEM	ABA	−0.63	0.367		MEM	ABA	0.80	0.200		TZP	EN-	−0.74	0.262
**ETP (↑)**	ETP	KPN	0.63	0.367	**ETP (↑)**	ETP	KPN	0.83	0.166	**ETP (↔)**	ETP	KPN	−0.77	0.225
	ETP	EN-	0.89	0.105		ETP	EN-	0.00	1.000		ETP	EN-	0.77	0.225
	IPM	PAE	0.60	0.400		IPM	PAE	0.95	0.051		IPM	PAE	−0.34	0.656
	MEM	PAE	0.60	0.400		MEM	PAE	0.63	0.367		MEM	PAE	0.74	0.262
**CAZ (↔)**	MEM	ABA	−0.93	0.007	**CAZ (↔)**	MEM	ABA	0.60	0.400	**CAZ (↓)**	MEM	ABA	−0.34	0.685
	IPM	ABA	−0.93	0.007		IPM	ABA	0.60	0.400		IPM	ABA	−0.30	0.699
	MEM	PAE	0.80	0.200		MEM	PAE	0.97	0.031		MEM	PAE	0.21	0.789
	IPM	PAE	0.80	0.200		IPM	PAE	0.50	0.497		IPM	PAE	−0.27	0.722
**CIP (↔)**	CRO	EN-	0.20	0.800	**CIP (↔)**	CRO	EN-	0.88	0.115	**CIP (↓)**	CRO	EN-	−0.80	0.200
	CAZ	KPN	0.80	0.200		CAZ	KPN	−0.05	0.953		CAZ	KPN	0.20	0.800
	CAZ	EN-	0.40	0.600		CAZ	EN-	0.63	0.367		CAZ	EN-	−0.60	0.400
	MEM	EN-	−0.89	0.105		TZP	EN-	0.83	0.171		TZP	EN-	−0.95	0.051
**CIP (↔)**	OXA	SAU	−0.20	0.800	**CIP (↔)**	OXA	SAU	−0.80	0.200	**CIP (↓)**	OXA	SAU	−0.75	0.242
	IPM	PAE	0.80	0.200		MEM	PAE	0.80	0.200		MEM	PAE	0.21	0.789
	AMK	PAE	0.40	0.600		AMK	PAE	0.80	0.200		AMK	PAE	0.80	0.200
	IPM	ABA	−0.89	0.041		MEM	ABA	0.80	0.200		MEM	ABA	−0.30	0.695
	MEM	ABA	−0.89	0.041		TZP	EN-	0.97	0.031		TZP	EN-	−0.74	0.262
**AMK (↔)**	AMK	PAE	0.63	0.367	**AMK (↓)**	AMK	PAE	0.13	0.868	**AMK (↓)**	AMK	PAE	0.31	0.688
	AMK	KPN	0.63	0.367		AMK	KPN	−0.83	0.163		AMK	KPN	−0.67	0.327
	AMK	EN-	0.32	0.683		AMK	EN-	0.32	0.683		AMK	EN-	−0.57	0.427
**IPM (↔)**	IPM	KPN	−0.95	0.051	**IPM (↔)**	IPM	KPN	−0.83	0.167	**MEM (↔)**	MEM	KPN	−0.56	0.436
	IPM	PAE	0.40	0.600		IPM	PAE	−0.32	0.683		TZP	EN-	−0.21	0.789
	MEM	KPN	−0.95	0.051		MEM	KPN	−0.81	0.183		TZP	KPN	−0.80	0.200
	TZP	KPN	−0.20	0.800		TZP	KPN	−0.32	0.683		MEM	PAE	0.74	0.262
	TZP	EN-	0.20	0.800		TZP	EN-	0.32	0.683		MEM	ABA	−0.01	0.847
**CLI (↔)**	OXA	SAU	0.40	0.600	**CLI (↔)**	OXA	SAU	−0.40	0.600	**CLI (↓)**	OXA	SAU	−0.94	0.051
	CLI	SAU	0.40	0.600		CLI	SAU	−0.40	0.600		CLI	SAU	−0.80	0.200
**SAM (↔)**	MEM	ABA	−0.77	0.225	**SAM (↔)**	MEM	ABA	0.32	0.683	**SAM (↓)**	MEM	ABA	−0.31	0.683
	IPM	ABA	−0.77	0.225		IPM	ABA	0.32	0.683		IPM	ABA	−0.33	0.665
**VAN (↔)**	VAN	EFM	−0.20	0.800	**VAN (↔)**	VAN	EFM	0.95	0.051	**VAN (↓)**	VAN	EFM	−0.25	0.746

Abbreviations: SAU = *Staphylococcus aureus*, EFM = *Enterococcus faecium*, KPN = *Klebsiella pneumoniae*, PAE = *Pseudomonas aeruginosa*, ABA = *Acinetobacter baumannii*, EN**-** =*Enterobacter* spp. AMK = amikacin, CAZ = ceftazidime, CIP = ciprofloxacin, CLI = clindamycin, CRO= ceftriaxone, ETP = ertapenem, IPM = imipenem, MEM = meropenem, OXA = oxacillin, SAM =ampicillin/sulbactam, TZP = piperacillin/tazobactam, VAN = vancomycin. R = Spearman’s rank correlation coefficient. *P* = statistical significance. Trend: ↑ = increasing, ↔ = stable, ↓ = decreasing.

## Data Availability

The data supporting the reported results are available from the corresponding author on reasonable request.

## Supplementary Materials

The following are available online at https://www.mdpi.com/article/10.3390/antibiotics10101221/s1, Table S1: Percentages of antimicrobial resistance of ESKAPE pathogens and their evolution between the period 2015 and 2018 by areas, Table S2: Consumption and percentage of change of antimicrobials (DDD/100 bed-days) during the period 2015–2018 by area.
