# Supplementary material for: Antibiotic Consumption and Its Relationship with Bacterial Resistance Profiles in ESKAPE Pathogens in a Peruvian Hospital
Source: Antibiotics (Basel). 2021 Oct 8;10(10):1221. doi: 10.3390/antibiotics10101221 (PMC8532675; doi:10.3390/antibiotics10101221)
Supplement: Supplementary file 1 [file antibiotics-10-01221-s001.zip › antibiotics-1391381-supplementary.pdf]

Table S1. Percentages of antimicrobial resistance of ESKAPE pathogens and their evolution between the period 2015-2018 by areas

**Area**			**Surgical ward**					**Medical wards**					**ICU**				
**Germ**	**Year**		**2015**	**2016**	**2017**	**2018**	***p***	**2015**	**2016**	**2017**	**2018**	***p***	**2015**	**2016**	**2017**	**2018**	***p***
**SAU**	**Number of isolates**		**24**	**31**	**31**	**22**		**43**	**59**	**92**	**50**		**13**	**20**	**41**	**42**	
	**ATB**	**OXA**	56.0	54.8	71.0	54.5	1.000	65.0	45.8	45.7	44.0	0.067	66.7	65.0	78.0	83.3	0.475
		**CIP**	54.0	67.8	64.5	63.6	0.725	43.0	61.0	47.8	42.0	1.000	66.7	65.0	82.9	78.6	0.750
		**CLI**	65.0	61.3	80.6	60.0	0.821	67.0	64.4	62.0	71.1	0.802	100.0	60.0	95.1	83.3	0.272
**EFM**	**N**		**17**	**34**	**39**	**20**		**24**	**78**	**35**	**29**		**3**	**13**	**34**	**25**	
	**ATB**	**VAN**	54.0	58.8	66.7	60.0	0.921	70.0	68.0	57.1	58.6	0.525	33.3	92.3	79.4	52.0	1.000
		**CIP**	94.0	97.1	97.5	96.0	1.000	96.0	98.7	94.3	100.0	0.923	100.0	100.0	100.0	100.0	-
**KPN**	**Number of isolates**		**87**	**137**	**78**	**66**		**115**	**127**	**71**	**149**		**78**	**59**	**62**	**125**	
	**ATB**	**ESBL %**	66.8	65.7	69.2	87.9	0.005	66.8	67.0	70.4	81.2	0.012	65.3	78.0	80.6	74.6	0.224
		**CAZ**	68.1	62.8	60.3	33.3	<0.001	72.3	64.0	61.9	77.2	0.431	72.1	74.6	69.4	72.5	1.000
		**IPM**	0	0	1.3	30.3	<0.001	0	0	2.8	26.2	<0.001	0	0	0	29.6	<0.001
		**CIP**	65.0	70.1	73.1	80.3	0.067	66.3	68.0	73.2	77.2	0.063	65.2	54.3	70.9	71.8	0.402
		**AMK**	2.2	5.8	6.4	10.6	0.069	1.0	2.0	7.0	6.0	0.063	3	0	3.2	7.0	0.271
		**TZP**	13.0	12.4	11.6	51.5	<0.001	15.0	18.8	15.5	45.6	<0.001	22.0	27.1	21.0	43.7	0.002
		**ETP**	0	0	2.6	31.8	<0.001	0	0	2.8	27.5	<0.001	0	0	0	30.3	<0.001
		**MEM**	0	0	1.3	31.8	<0.001	0	0	2.8	25.5	<0.001	0	0	0	29.6	<0.001
**PAE**	**Number of isolates**		**75**	**126**	**52**	**108**		**31**	**154**	**89**	**155**		**75**	**114**	**107**	**126**	
	**ATB**	**CAZ**	66.0	65.8	48.1	63.0	0.721	55.6	54.7	47.2	65.1	0.376	66.4	72.8	73.9	65.9	1.000
		**TZP**	40.1	58.7	34.6	45.4	0.568	37.2	48.8	33.7	51.0	0.293	67.0	63.2	56.1	28.6	<0.001
		**IPM**	76.0	76.9	48.1	72.2	0.689	56.4	64.6	53.9	73.5	0.062	82.7	83.3	85.0	77.0	0.436
		**MEM**	51.0	73.0	48.1	59.3	0.317	58.2	60.2	48.3	57.4	1.000	79.3	79.8	85.0	65.9	0.077
		**CIP**	66.0	68.3	61.5	65.8	1.000	55.4	58.3	56.2	60.6	0.688	64.0	70.2	74.7	57.1	0.418
		**AMK**	25.0	57.9	44.2	46.3	0.007	43.0	48.0	40.4	34.9	0.584	65.0	62.3	60.7	34.9	<0.001
**ABA**	**Number of isolates**		**15**	**10**	**28**	**25**		**17**	**21**	**13**	**81**		**43**	**84**	**26**	**106**	
	**ATB**	**IPM**	91.0	90.0	91.0	92.0	1.000	88.0	88.0	90.0	86.4	1.000	90.0	89.0	91.0	92.5	0.980
		**MEM**	91.0	90.0	91.0	92.0	1.000	86.0	84.5	82.5	82.7	0.842	88.0	86.0	78.0	95.3	0.244
		**CAZ**	94.0	95.0	98.0	96.0	1.000	95.0	88.0	90.0	87.7	0.730	97.0	95.0	94.3	93.4	0.517
		**CIP**	92.0	95.0	96.0	92.0	1.000	94.0	90.0	91.0	87.7	1.000	90.0	87.0	88.0	93.4	0.822
		**AMK**	64.0	70.0	66.0	68.0	1.000	56.0	57.0	54.0	58.0	0.730	70.0	65.0	66.0	72.6	0.879
		**SAM**	75.0	82.0	80.0	84.0	0.683	60.0	50.0	56.0	65.4	0.811	78.0	78.0	81.0	83.0	0.740
**EN-**	**Number of isolates**		**14**	**25**	**17**	**18**		**25**	**44**	**17**	**27**		**4**	**7**	**14**	**29**	
	**ATB**	**CAZ**	55.0	60.0	76.5	55.5	1.000	54.0	61.4	53.0	51.8	0.983	30.0	28.6	64.2	41.4	0.934
		**TZP**	23.0	28.0	23.5	27.8	1.000	21.0	31.9	20.0	25.9	0.859	0	0	50.0	3.4	1.000
		**IPM**	0	0	0	0	-	0	0	0	0	-	0	0	14.3	0	-
		**CIP**	54.0	60.0	88.2	66.7	0.854	43.0	54.5	47.1	55.5	0.579	15.0	14.3	42.8	17.2	1.000
		**ETP**	0	4.2	0	5.6	1.000	0	13.6	0	0	-	0	0	21.4	0	-
		**MEM**	0	0	0	0	-	0	0	0	0	-	0	0	14.3	0	-
		**CRO**	65.0	72.0	76.5	66.7	1.000	60.0	65.9	58.8	59.3	1.000	27.0	28.6	71.4	48.3	0.733
		**AMK**	3.0	24.0	17.6	11.2	0.581	5.0	18.2	5.9	7.4	1.000	1.0	0	14.2	0	-

* Abbreviations: SAU=*Staphylococcus aureus*, EFM=*Enterococcus faecium*, KPN=*Klebsiella pneumoniae*, PAE=*Pseudomonas aeruginosa*, ABA=*Acinetobacter baumannii*, EN- =*Enterobacter spp*. ** ATB=antimicrobial. AMK=amikacin, CAZ=ceftazidime, CIP=ciprofloxacin, CLI=clindamycin, CRO= ceftriaxone, ETP=ertapenem, IPM=imipenem, MEM=meropenem, OXA=oxacillin, SAM =ampicillin/sulbactam, TZP=piperacillin/tazobactam, VAN=vancomycin. ICU=intensive care unit. ESBL=Extended spectrum Beta-lactamase. To calculate the p-value, the difference in proportions test was applied considering the first and last year of study and $p \leq 0.05$ was considered statistically significant.

Table S2. Consumption and percentage of change of antimicrobials (DDD / 100 bed-days) during the period 2015-2018 by area.

ATB	Surgical ward						
	DDD/100 bed-days				Mean	% Change	SD
	2015	2016	2017	2018			
SAM	0.22	0.33	0.21	0.16	0.23	-26.65	0.07
CAZ	1.21	1.99	0.80	0.86	1.21	-29.22	0.55
CRO	16.67	23.69	10.10	12.07	15.63	-27.60	6.03
CIP	3.20	4.60	1.69	3.04	3.13	-5.01	1.19
COL	0.09	0.26	0.30	0.42	0.27	349.49	0.13
IPM	2.75	4.46	2.46	1.96	2.91	-28.69	1.09
LNZ	0.26	0.40	0.27	0.52	0.37	98.61	0.12
MEM	1.36	2.47	1.40	2.13	1.84	56.99	0.55
TZP	1.03	2.44	1.03	1.84	1.58	79.10	0.68
VAN	2.96	4.79	2.69	3.34	3.45	12.85	0.94
OXA	0.38	0.45	0.36	0.51	0.42	32.49	0.07
AMK	3.31	4.78	2.67	3.31	3.52	0.06	0.90
CLI	5.71	9.13	5.36	4.80	6.25	-16.02	1.96
ETP	0.66	1.30	0.88	1.73	1.14	163.72	0.47
TIG	0.16	0.10	0.18	0.51	0.24	218.22	0.18
ATB	Medical wards						
	DDD/100 bed-days				Mean	% Change	SD
	2015	2016	2017	2018			
SAM	0.83	0.75	0.87	1.00	0.86	20.58	0.10
CAZ	4.45	7.88	4.46	4.66	5.36	4.91	1.68
CRO	7.16	9.86	5.50	5.06	6.89	-29.32	2.17
CIP	3.71	6.97	3.58	4.00	4.57	7.86	1.61
COL	0.17	0.33	0.34	0.60	0.36	259.43	0.18
IPM	9.95	13.56	9.91	9.23	10.66	-7.23	1.96
LNZ	0.93	1.15	0.75	0.97	0.95	4.98	0.16
MEM	1.99	3.27	2.79	4.20	3.06	110.57	0.92
TZP	1.58	2.90	3.03	3.89	2.85	146.12	0.95
VAN	7.15	11.21	8.13	7.86	8.59	10.04	1.80
OXA	3.37	1.75	2.76	1.59	2.37	-52.72	0.84
AMK	4.21	4.94	2.59	2.10	3.46	-50.18	1.34
CLI	4.55	7.04	4.01	4.46	5.02	-2.04	1.37
ETP	0.91	1.37	1.36	1.86	1.37	104.58	0.39
TIG	0.12	0.17	0.21	0.44	0.23	258.84	0.14
ATB	ICU						
	DDD/100 bed-days				Mean	% Change	DE
	2015	2016	2017	2018			
SAM	0.67	0.41	0.34	0.29	0.42	-56.95	0.17
CAZ	5.47	7.15	4.22	2.02	4.72	-63.04	2.16
CRO	6.21	7.74	3.15	3.44	5.14	-44.53	2.22
CIP	1.42	2.25	1.23	0.35	1.31	-75.06	0.78
COL	1.11	2.17	1.41	1.58	1.57	42.14	0.44
IPM	6.05	7.08	5.66	2.20	5.25	-63.65	2.12
LNZ	0.80	1.88	2.37	1.80	1.71	125.42	0.66
MEM	7.43	12.04	9.87	7.17	9.13	-3.49	2.29
TZP	2.28	4.22	1.57	2.30	2.59	0.61	1.14
VAN	10.03	13.86	8.03	4.74	9.16	-52.76	3.82
OXA	0.36	0.69	0.88	0.66	0.65	80.41	0.21
AMK	3.97	4.41	2.94	1.96	3.32	-50.63	1.10
CLI	3.14	3.62	1.72	1.37	2.46	-56.31	1.08
ETP	0.45	1.05	0.45	0.31	0.56	-31.08	0.33
TIG	0.62	0.77	0.56	0.95	0.72	52.92	0.17

* Abbreviations: ATB=antimicrobial. SD= standard deviation. AMK=amikacin, CAZ=ceftazidime, CIP=ciprofloxacin, CLI=clindamycin, COL=colistin, CRO= ceftriaxone, ETP=ertapenem, IPM=imipenem, LNZ=linezolid, MEM=meropenem, OXA=oxacillin, SAM =ampicillin/sulbactam, TZP=piperacillin/tazobactam, VAN=vancomycin, TIG=tigecycline. ICU=intensive care unit. To calculate the p-value, the difference in proportions test was applied considering the first and last year of study and $p \leq 0.05$ was considered statistically significant. The percentage of change was calculated by subtracting the consumption data (DDD / 100 beds-days) from 2018 compared to 2015, divided by the consumption in the first-year of study, and multiplying the result by 100
